# Supplementary material for: Molecular basis for assembly of the shieldin complex and its implications for NHEJ
Source: Nat Commun. 2020 Apr 24;11:1972. doi: 10.1038/s41467-020-15879-5 (PMC7181697; doi:10.1038/s41467-020-15879-5)
Supplement: Supplementary file 1 — Supplementary Information [file 41467_2020_15879_MOESM1_ESM.pdf]

Supplementary Information

Molecular basis for assembly of the shieldin complex and its implications for NHEJ

Liang et al.

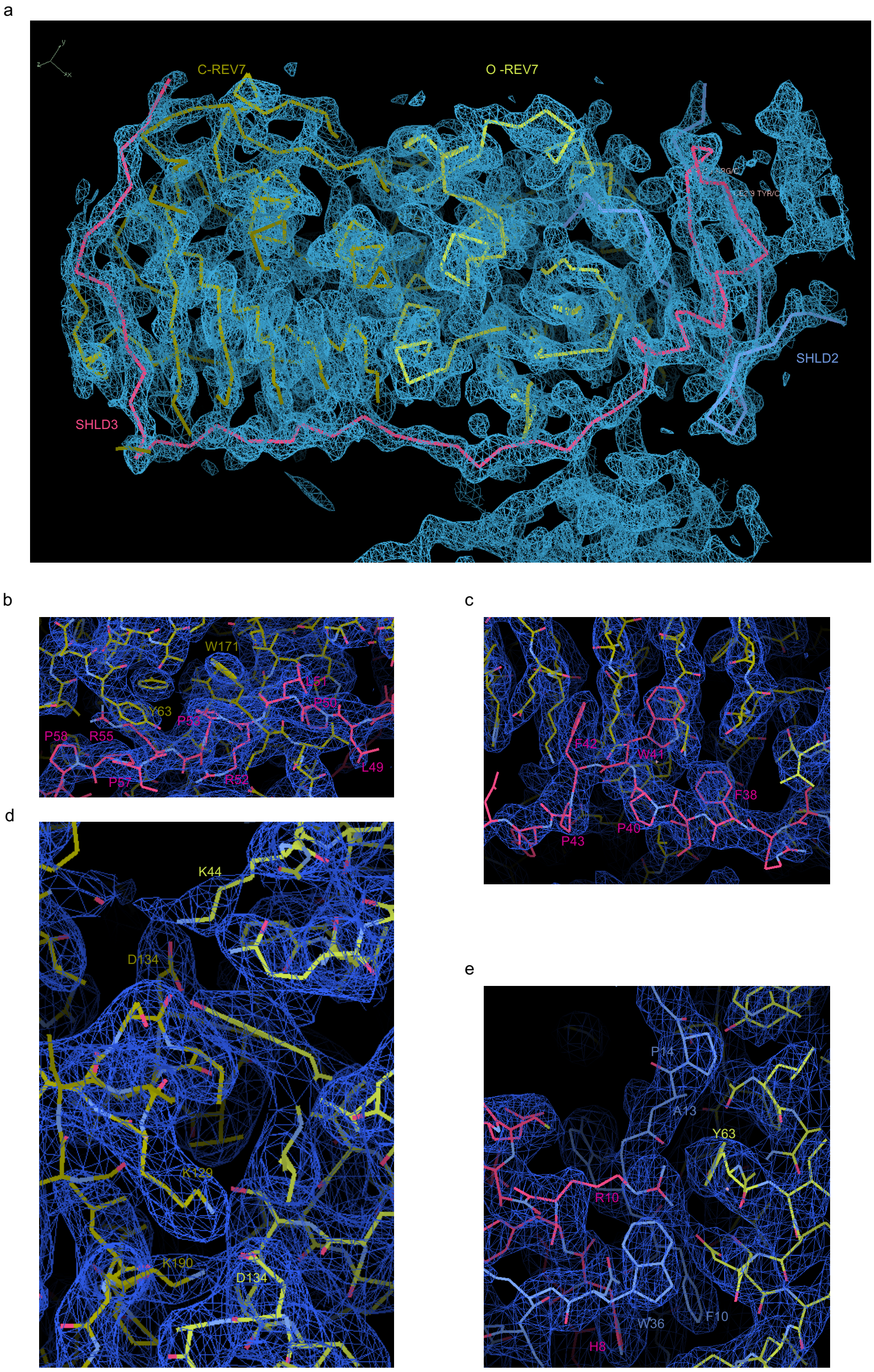

Supplementary Figure 1. **Overview of the 2Fo-Fc electron density map of the SHLD3-REV7-SHLD2 complex and local density at regions that are important for the formation of the complex.** The superimposed magenta, dark yellow, light yellow and blue Cα chains are that of SHLD3, C-REV7, O-REV7 and SHLD2, respectively. **a**, Overview of the 2Fo-Fc electron density map of the SHLD3-REV7-SHLD2 complex. The map was contoured at 1.5σ. **b-e**, Sections of the 2Fo-Fc electron density map

surrounding some important regions contoured at 1.3σ allows unambiguous placement of protein sidechains. RBM_2 of SHLD3 (**b**). The FXPWFP motif of SHLD3 (**c**). Some important residues at the conformational dimer interface (**d**). The interface between O-REV7 and SHLD2 centered around Tyr63 of O-REV7 (**e**). C-REV7 residues were shown with the carbon bonds colored dark yellow, while light yellow, red and blue carbon bonds indicate that of O-REV7, SHLD3 and SHLD2, respectively. Red and blue bonds represent that of oxygen and nitrogen, respectively.

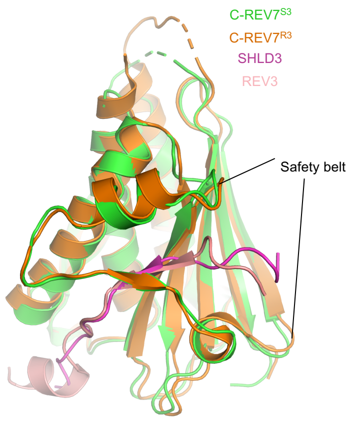

Supplementary Figure 2. **Structural alignment of C-REV7-SHLD3 and C-REV7-REV3.**

Structural superimposition of C-REV7 (green) –SHLD3 (magenta) and C-REV7 (orange) –REV3 (salmon) (PDB: 3VU7). C-REV7^{S3} represents SHLD3 bound C-REV7, and C-REV7^{R3} represents REV3 bound C-REV7. The superimposition was done by align C-REV7^{S3} to C-REV7^{R3} with an RMSD (root mean square deviations) of 0.731 Å.

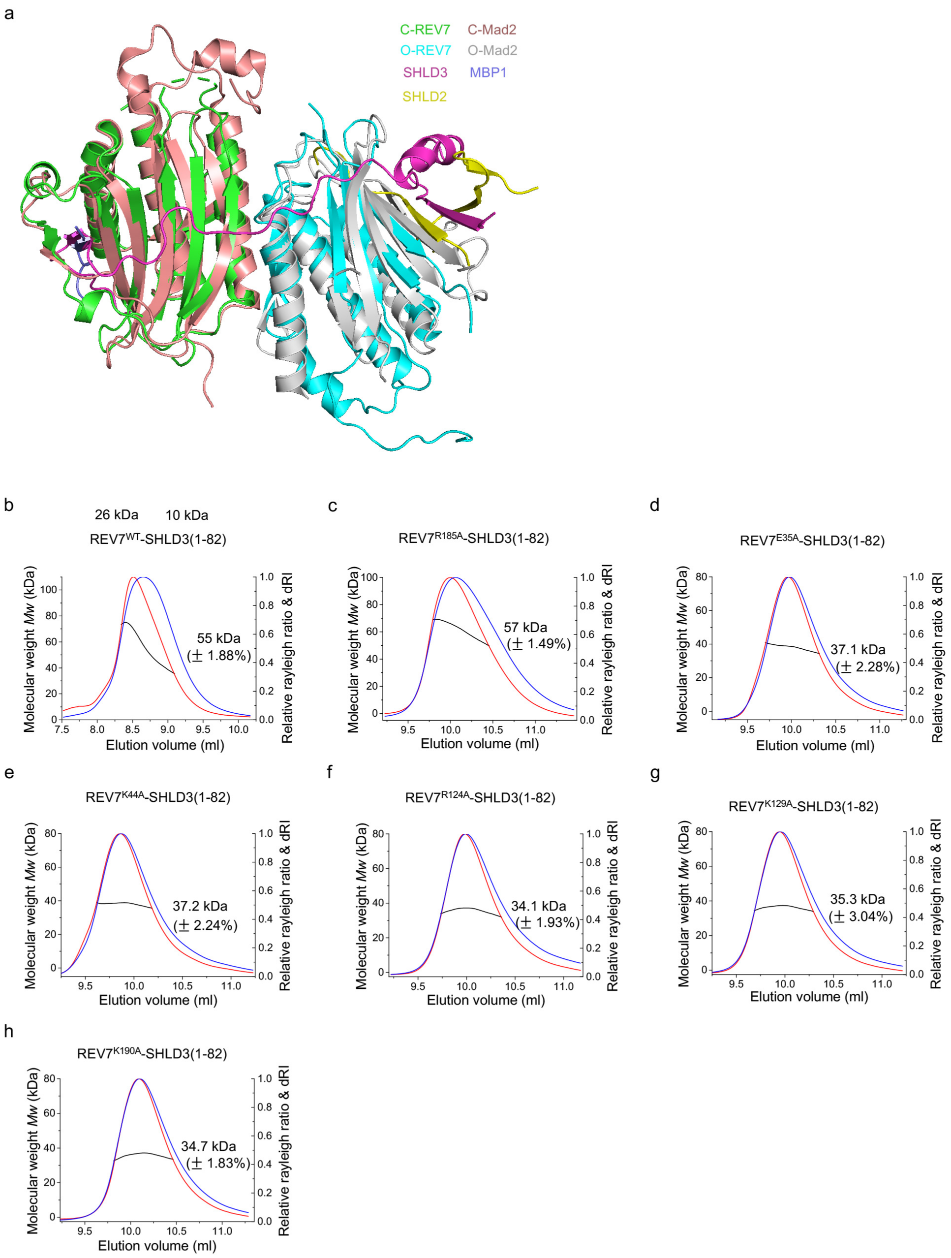

Supplementary Figure 3. **Structural alignment of C-REV7-O-REV7 and C-Mad2-O-Mad2 and molecular weights measured by SEC-MALS.**

a, Structural superimposition of C-REV7 (green)-O-REV7 (cyan) and C-Mad2 (salmon) -O-Mad2 (gray) (PDB: 2V64). The superimposition was done by align C-REV7 to C-Mad2 with an RMSD of 1.27 Å. **b**, The calculated masses for recombinant His-REV7 and SHLD3(1-82) are 26.2 kDa and 9.4 kDa, respectively. The calculated masses for SHLD3(1-82)-C-REV7-O-REV7 and SHLD3(1-82)-C-REV7 are 61.8 kDa and 35.6 kDa, respectively. WT: wild type. Molecular weight of REV7^{WT}-SHLD3(1-82) is 55 kDa, which indicates the sample was not homogeneous, some were one SHLD3(1-82) with two REV7 molecules and others were one SHLD3(1-82) with one REV7 molecule.

c, Arg185 is a residue that does not locate in the interface, hence REV7^{R185A} behaved like REV7^{WT}. **d-h**, Mutations of residues that locate at the dimer interface abolished SHLD3 mediated REV7 conformational dimer formation and form homogeneous C-REV7-SHLD3(1-82) heterodimer. The calculated mass for C-REV7-SHLD3(1-82) is 35.6 kDa. The fitted errors obtained from the data analysis software are showed in the brackets.

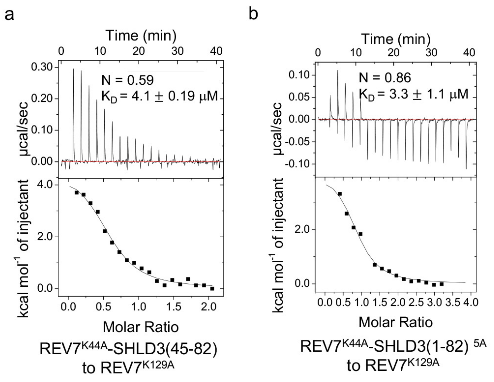

Supplementary Figure 4. **a-b, ITC measurements of binding affinity between REV7^{K44A}-SHLD3(45-82) or REV7^{K44A}-SHLD3(1-82)^{5A} and REV7^{K129A}**. The calculated N and K_D are indicated as described in Fig. 2d. Source data are provided as a Source Data file.

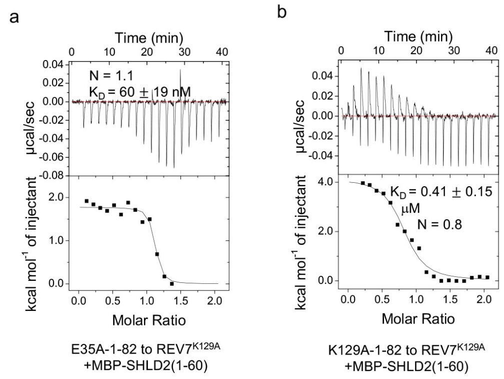

Supplementary Figure 5. **ITC measurements of binding affinity between REV7-SHLD3(1-82) mutants and REV7^{K129A} in the context of MBP-SHLD2(1-60).**

a, ITC measurement of the interaction between REV7^{E35A}-SHLD3(1-82) (E35A-1-82) and REV7^{K129A} in the context of MBP-SHLD2(1-60). Excessive MBP-SHLD2(1-60) was first incubated with REV7^{K129A}, then REV7^{E35A}-SHLD3(1-82) was titrated into the cell containing REV7^{E35A}-SHLD3(1-82) and MBP-SHLD2(1-60). **b**, ITC measurement of the interaction between REV7^{K129A}-SHLD3(1-82) (K129A-1-82) and REV7^{K129A} in the context of MBP-SHLD2(1-60). The titration was done as described in Supplementary Fig. 5a. The calculated N and K_D are indicated as described in Fig. 2d. Source data are provided as a Source Data file.

Figure S6

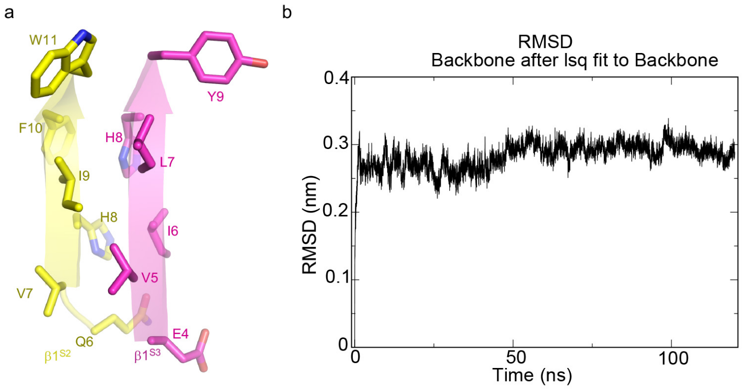

Supplementary Figure 6. **The N terminus of SHLD3 binds to O-REV7.**

a, Structural details of β1 of SHLD2 (β1^{S2}) and β1 of SHLD3 (β1^{S3}). **b**, Root mean square deviations (RMSD) of C-REV7-O-REV7-SHLD3(1-82) along the MD simulation.

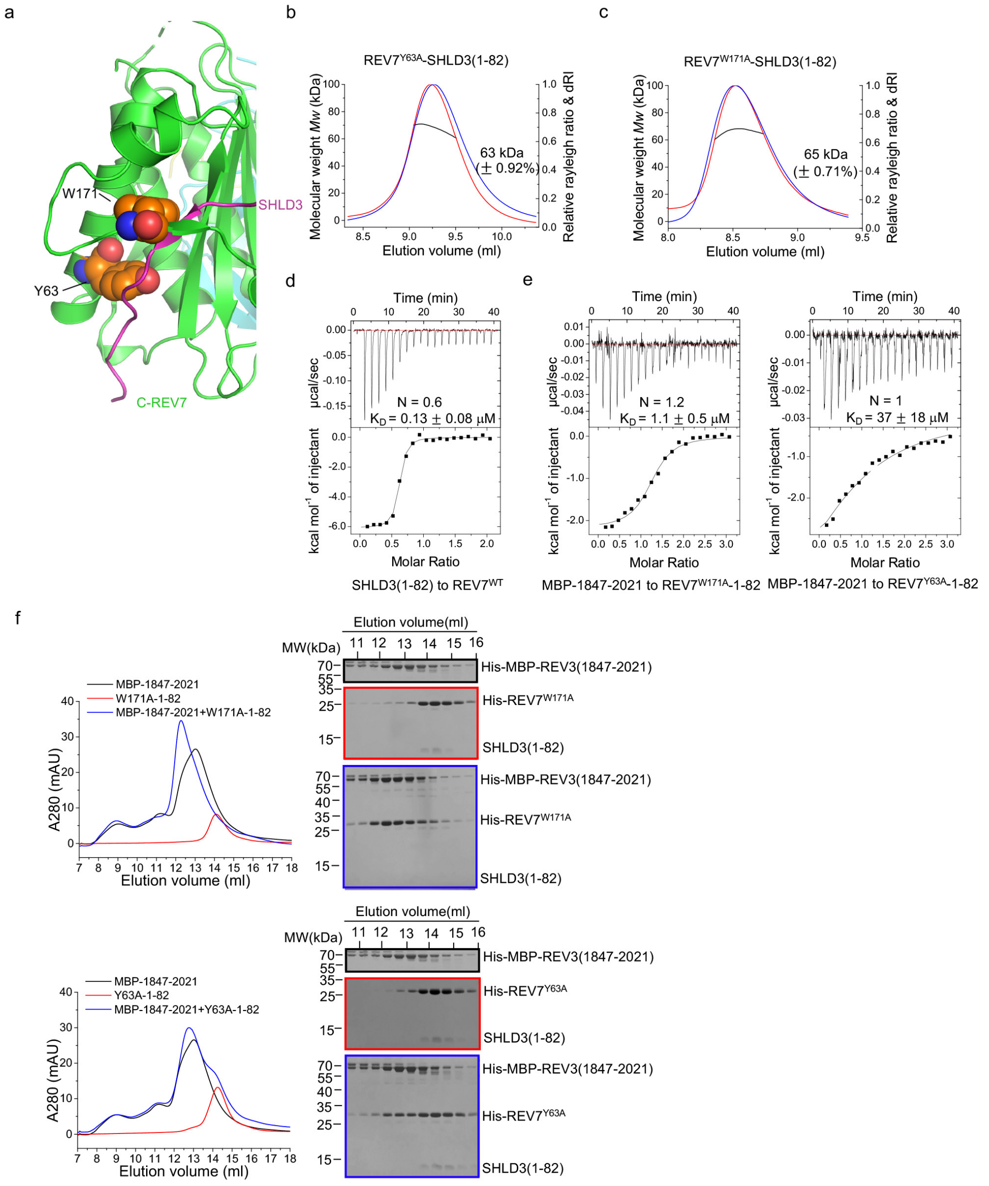

Supplementary Figure 7. **REV7^{Y63A}-SHLD3(1-82) shows impaired ability to interact with REV3 as compared with REV7^{W171A}-SHLD3(1-82).**

a, Structural details of C-REV7-SHLD3$_{RBM2}$ shown by cartoon model. C-REV7 is shown in green, O-REV7 is shown in cyan and SHLD3 is shown in magenta. Tyr63 and Trp171 of C-REV7 are shown in spheres model to highlight their interactions with SHLD3. Tyr63, Trp171 and β sheet of SHLD3 form a sandwich to stabilize the complex. **b,c**, Molecular weights measured by SEC-MALS. Molecular weights of REV7^{Y63A}-SHLD3(1-82) and REV7^{W171A}-SHLD3(1-82) are 63 kDa and 65 kDa, respectively, which correspond to one SHLD3(1-82) with two REV7 molecules, and indicate the samples were homogeneous C-REV7-O-REV7-SHLD3(1-82). The calculated N and K_D are indicated as described in Fig. 2d. **d,** ITC measurement of the interaction between

SHLD3(1-82) and REV7. SHLD3(1-82) binds tightly to REV7 with a nanomolar affinity (about 130 nM) at a ratio about 1:2. **e**, ITC measurements of the interaction between MBP-REV3(1847-2021) and REV7^{Y63A}-SHLD3(1-82) or REV7^{W171A}-SHLD3(1-82). The calculated N and K_D are indicated as described in Fig. 2d. **f,** Gel filtration profiles show the interaction between MBP-REV3(1847-2021) and REV7^{Y63A}-SHLD3(1-82) or REV7^{W171A}-SHLD3(1-82) in a Superdex200 Increase 10/300 SEC column. Y63A-1-82 is short for REV7^{Y63A}-SHLD3(1-82) complex, W171A-1-82 is short for REV7^{W171A}-SHLD3(1-82) complex, and MBP-1847-2021 is short for MBP-REV3(1847-2021). Since MBP-REV3(1847-2021) competes away SHLD3(1-82), and SHLD3(1-82) eluted at 17 ml, the bands for SHLD3(1-82) cannot be seen in supplementary Fig. 7f (top, blue panel). n = 2 biologically independent experiments. Source data are provided as a Source Data file.

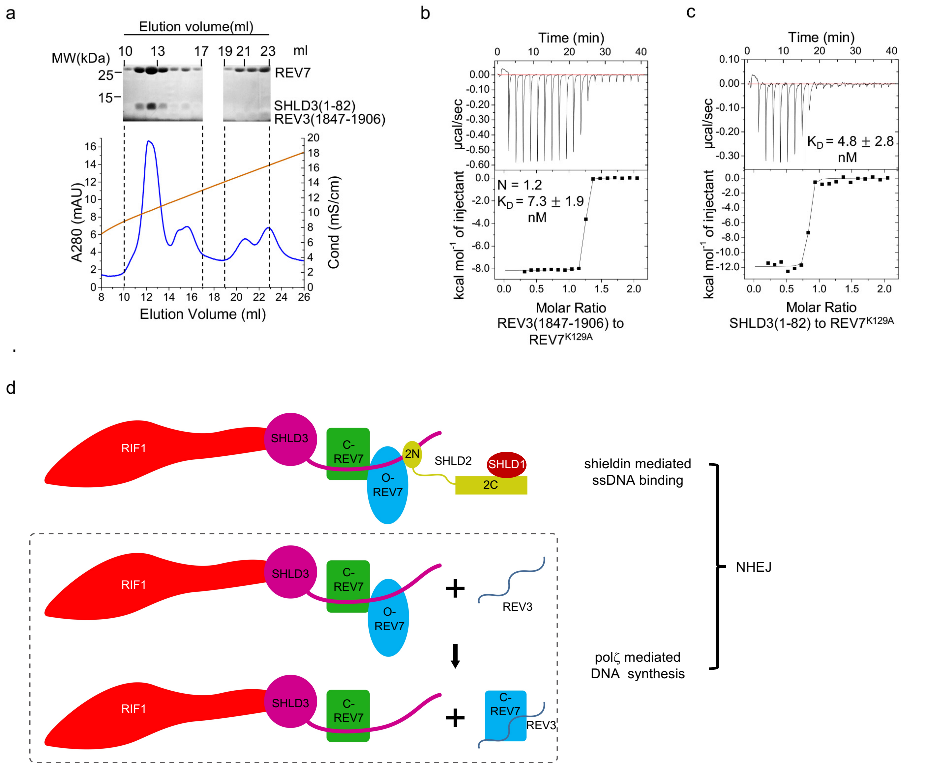

Supplementary Figure 8. REV3 and SHLD3 binds tightly to REV7^{K129A} with a similar binding affinity and molecular machinery of SHLD3 mediated REV7 conformational dimer in NHEJ.

a, Anion exchange analysis of the sample after titration in Fig. 8a in a Resource Q 1ml column. The blue line represents the absorbance at 280 nm and brown line represents the conductance. Fractions (1 ml each) were analyzed by SDS-PAGE and stained by Coomassie brilliant blue (the top panel), which shows two complexes (REV7-SHLD3 and REV7-REV3) formed and they were separated. n = 2 biologically independent experiments. **b**, ITC measurement of the interaction between REV3(1847-1906) and REV7^{K129A}. REV3(1847-1906) binds to REV7^{K129A} with a nanomolar affinity (K_D = 7.3 ± 1.9 nM). **c**, ITC measurement of the interaction between SHLD3(1-82) and REV7^{K129A}. SHLD3(1-82) binds to REV7^{K129A} with a nanomolar affinity (K_D = 4.8 ± 2.8 nM). The calculated N and K_D are indicated as described in Fig. 2d. Source data are provided as a Source Data file. **d**, Molecular machinery of SHLD3 mediated REV7 conformational dimer in NHEJ. Rectangle represents an REV7 in its closed state while Ellipse shows an open REV7. The C-REV7 and O-REV7 in SHLD3 mediated REV7 conformational dimer are colored in green and cyan, respectively. In addition to assembly shieldin complex with SHLD2 to bind ssDNA, the conformational dimer may act as a platform to coordinate various REV7 binding proteins, such as REV3.

Figures 5a-5c

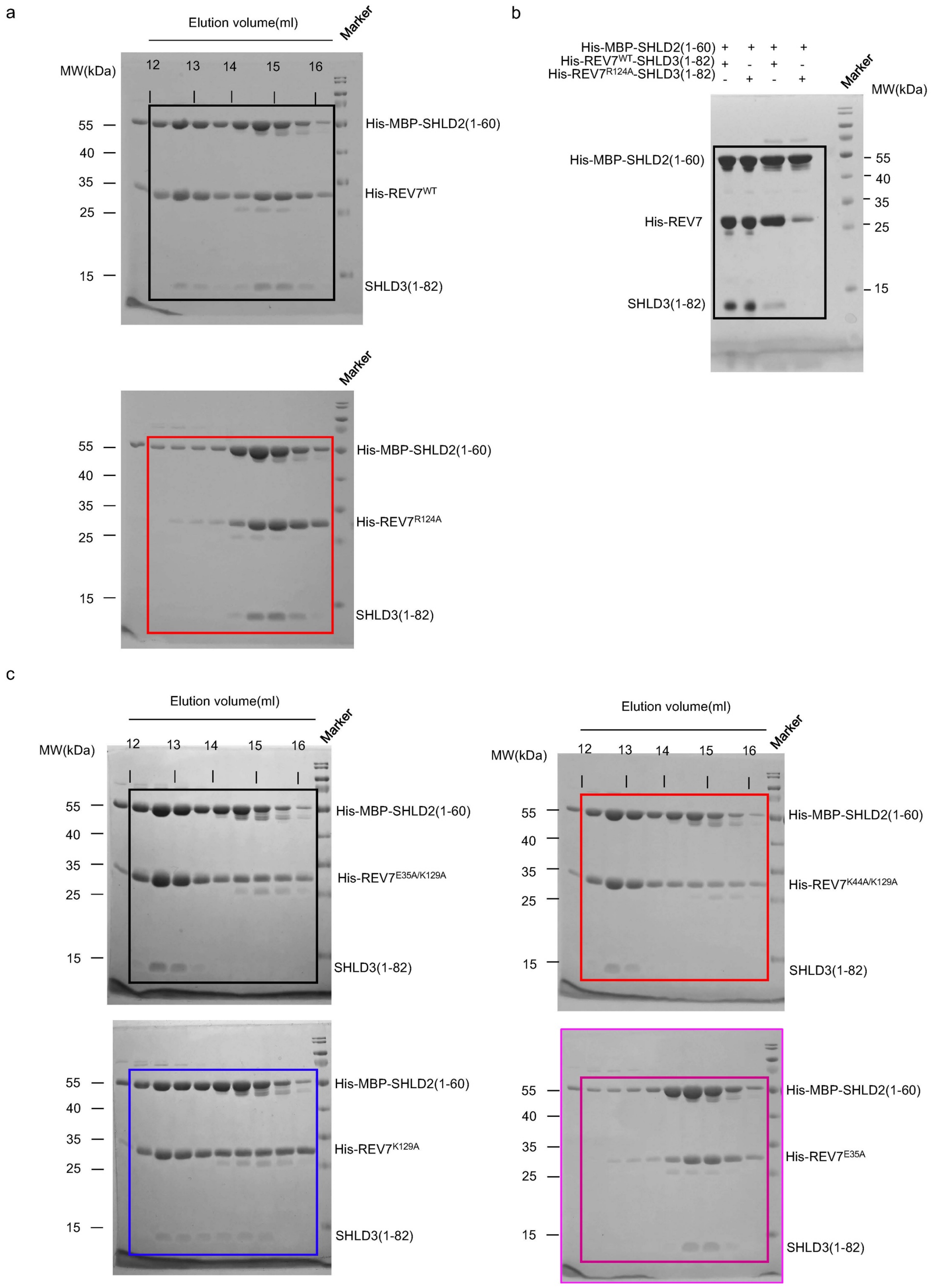

Supplementary Figure 9. Uncropped and unprocessed versions of gels and western blots presented in the main and supplementary figures.

Figure 5d

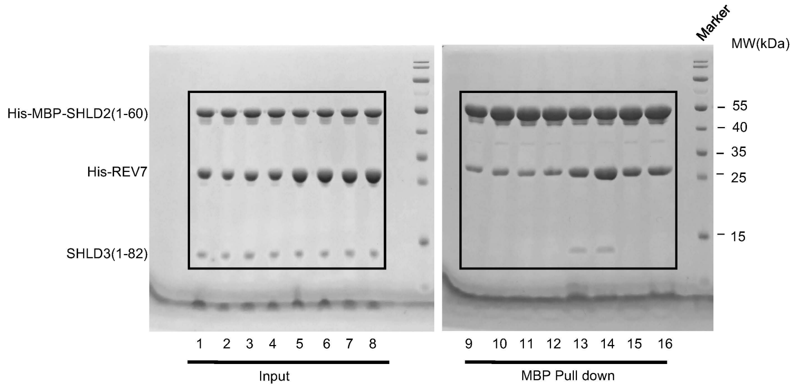

Figure 7b

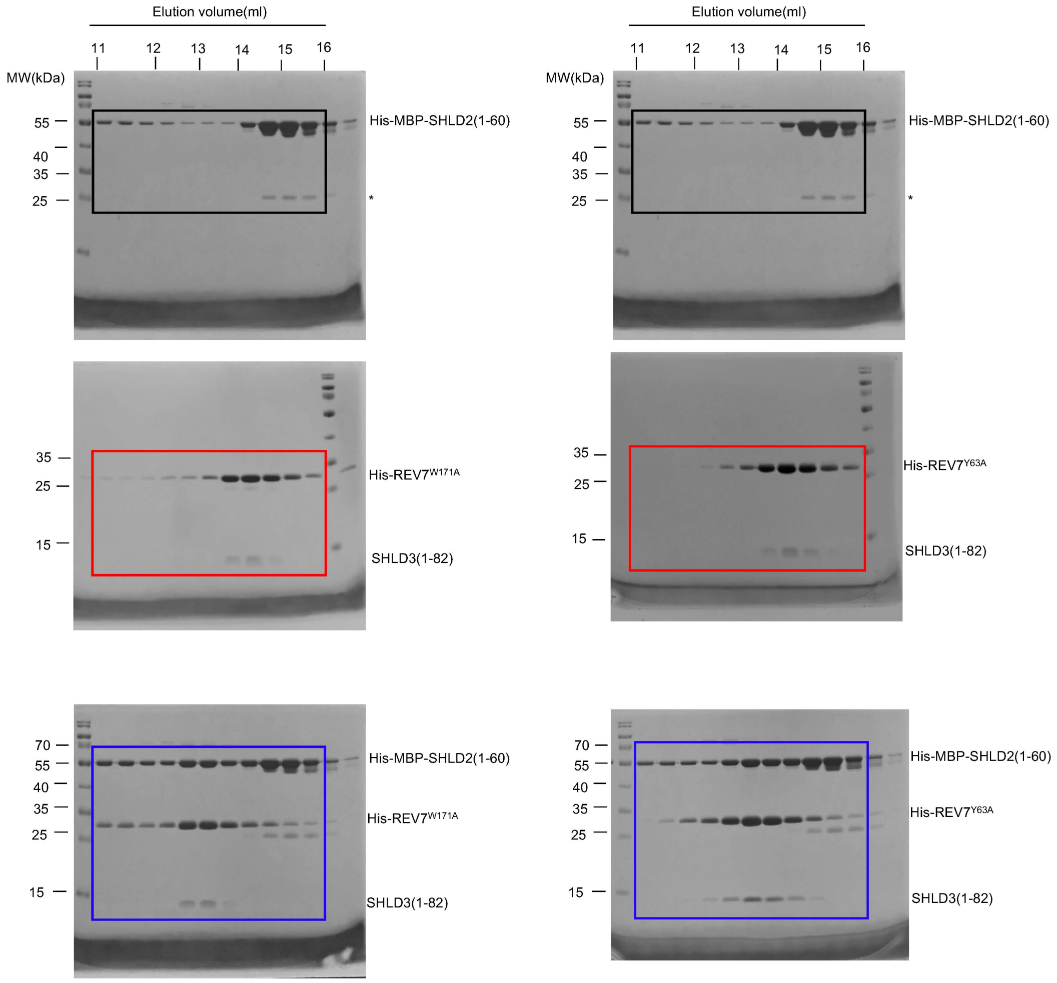

Supplementary Figure 9 (continued). Uncropped and unprocessed versions of gels and western blots presented in the main and supplementary figures.

Supplementary Figure 7f

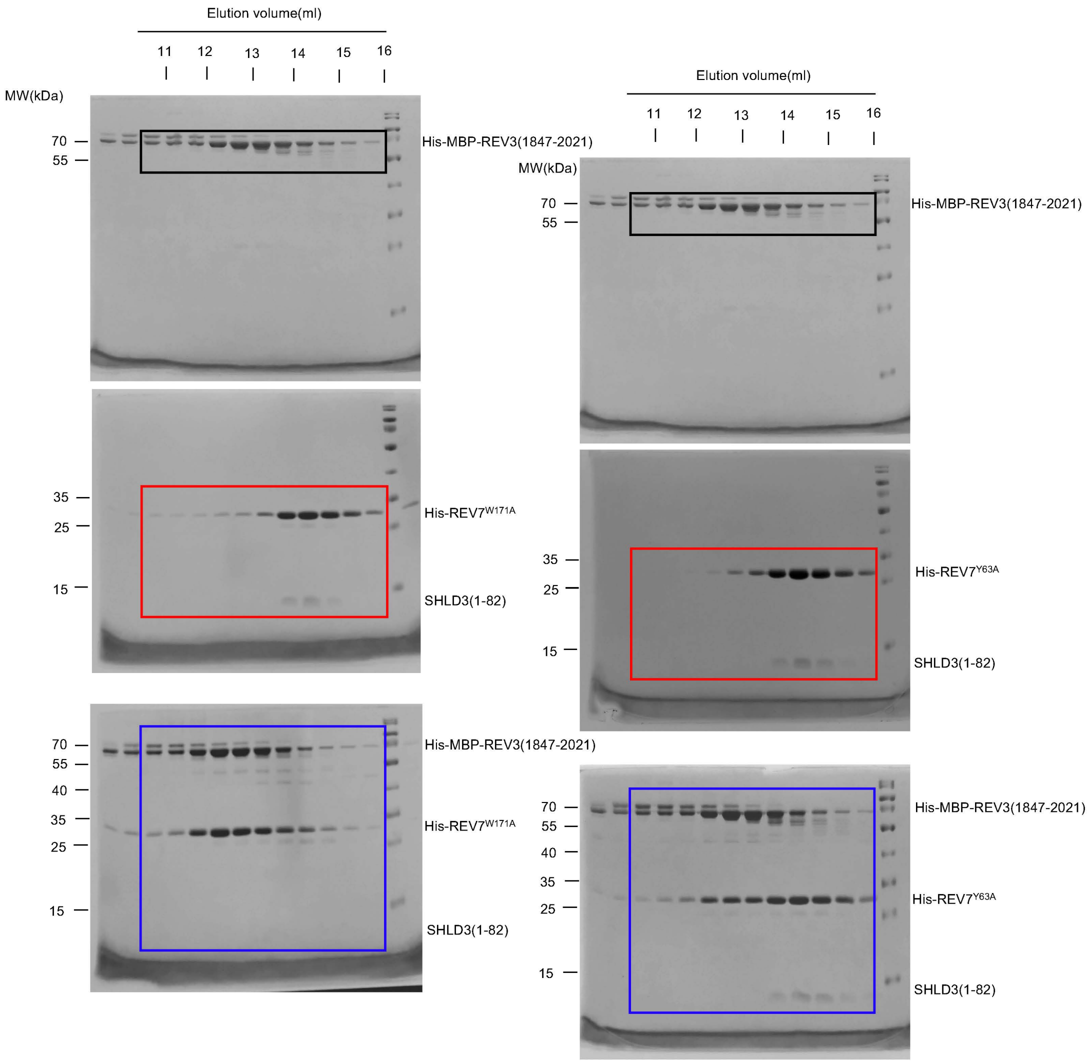

Supplementary Figure 9 (continued). Uncropped and unprocessed versions of gels and western blots presented in the main and supplementary figures.

Figure 8b

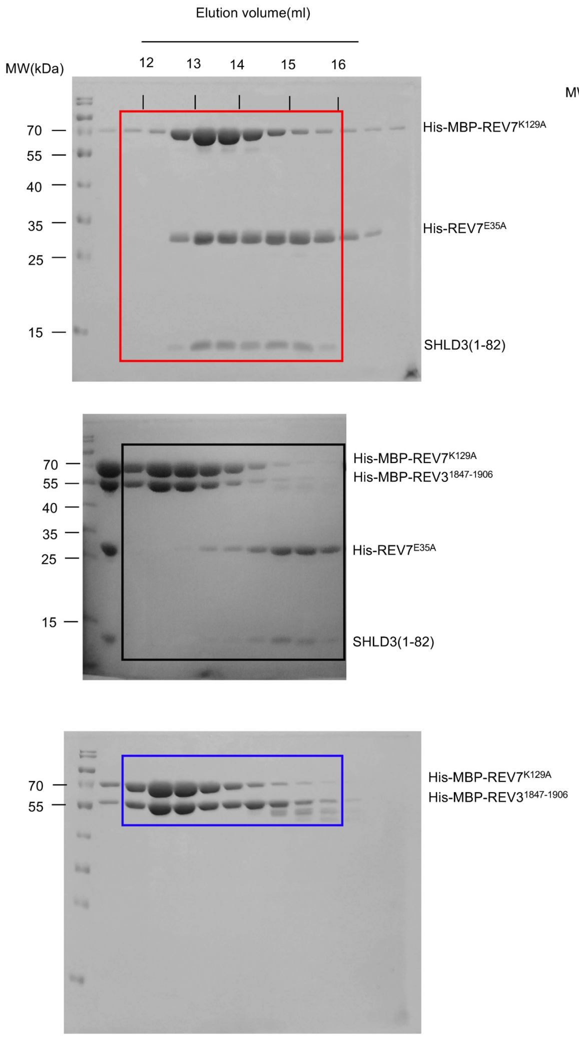

Figure 8d

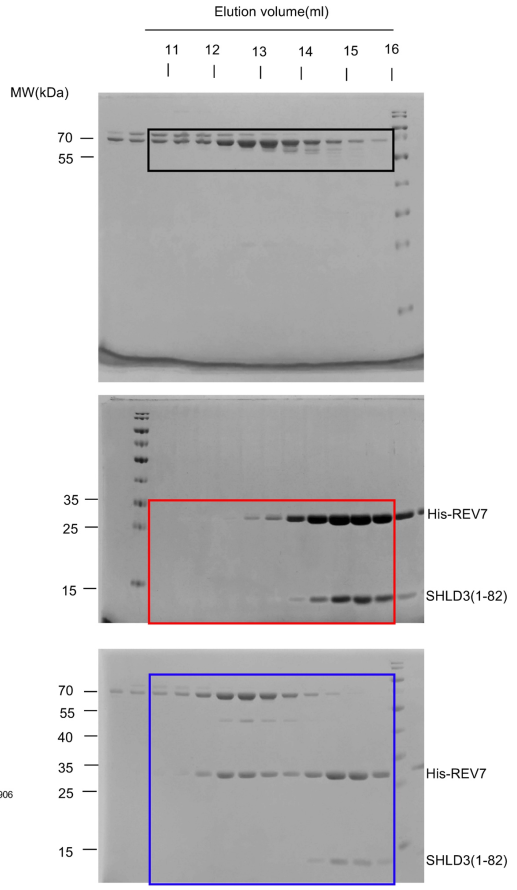

Supplementary Figure 8a

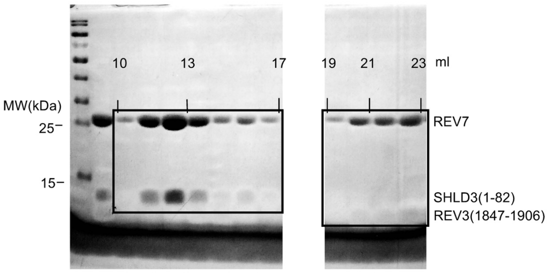

Supplementary Figure 9 (continued). Uncropped and unprocessed versions of gels and western blots presented in the main and supplementary figures.

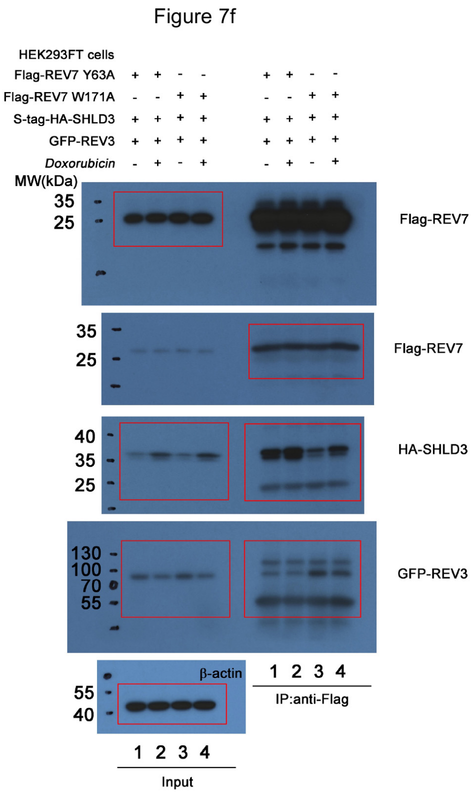

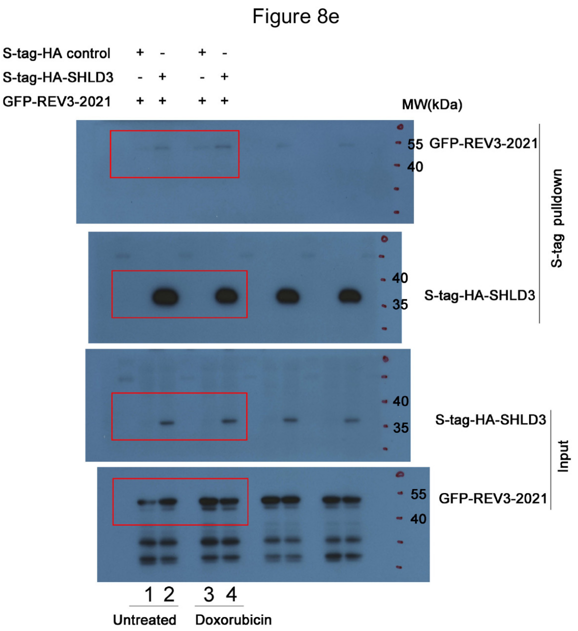

Supplementary Figure 9 (continued). Uncropped and unprocessed versions of gels and western blots presented in the main and supplementary figures.

Supplementary Table 1. The observed and calculated masses of the complexes measured by SEC-MALS.

	Observed Mass (kDa)	Calculated Mass (kDa)	Error (%)
$REV7^{WT}$-SHLD3(1-82)	55	36~62	ND
$REV7^{R185A}$-SHLD3(1-82)	57	36~62	ND
$REV7^{E35A}$-SHLD3(1-82)	37.1	36	3.1
$REV7^{K44A}$-SHLD3(1-82)	37.2	36	3.3
$REV7^{R124A}$-SHLD3(1-82)	34.1	36	-5.3
$REV7^{K129A}$-SHLD3(1-82)	35.3	36	-1.9
$REV7^{K190A}$-SHLD3(1-82)	34.7	36	-3.6
$REV7^{Y63A}$-SHLD3(1-82)	63	62	1.6
$REV7^{W171A}$-SHLD3(1-82)	65	62	4.8

Supplementary Table 2. Primers for constructing plasmids.

Vector	Forward primer (5'-3')	Backward primer (5'-3')
His-REV7	GGAATTCGATGACCACGCTCACACGAC	ATAAGAATGCGGCCGCTCAGCTGCCTTTATGAGCG
His-MBP-REV7	GGAATTCATGACCACGCTCACACGAC	ATAAGAATGCGGCCGCTCAGCTGCCTTTATGAGCG
Flag-REV7	CCGCTCGAGGCCACCATGGATTACAAGGATGACGACGATAAGACCACGCTCACACGAC	GCTCTAGATCAGCTGCCTTTATGAGCG
His-REV7-SHLD3(1-82)	GGAATTCCATATGACCACCGAAGTTATCCTGCACTACCGTCCGTGCGAAT	CCGCTCGAGTCAGTGAGATTTAGCGTCGTGTTCAGAGATGGTCAGGTACTGTTTAACG
His-REV7-SHLD3(1-64)	GGAATTCCATATGACCACCGAAGTTATCCTGCACTACCGTCCGTGCGAAT	CCGCTCGAGTCAAGCTTCTTCAGAGATAACCGGC
mCherry-SHLD3	CCGCTCGAGCTATGACCACCGAAGTTATCCT	CGGGATCCTTACATAGAGAAGATAACACCGTATT
REV7-SHLD3(28-82)	GGAATTCCATATGCAGGACTTCCCGACCCGTCCGCTGTCTCGTTTCATCCCGTGGTTCC	CCGCTCGAGTCAGTGAGATTTAGCGTCGTGTTCAGAGATGGTCAGGTACTGTTTAACG
SHLD3(1-82)-His	CATGCCATGGGCATGACCACCGAAGTTATCCTGC	CCGCTCGAGGTGAGATTTAGCGTCGTGTTCAG
S-tag-HA-SHLD3	CGGGATCCATGACCACCGAAGTTATCCT	ATAAGAATGCGGCCGCTTACATAGAGAAGATAACACCGTATT
SHLD2(1-60)	CATGCCATGGGCATGAGTGGAGGATCTCAAGTCC	CCGCTCGAGTCATTCAAGATTTTTGTGCTGTTTTTC
SHLD2(1-52)	CATGCCATGGGCATGAGTGGAGGATCTCAAGTCC	CCGCTCGAGTCAATCCTTCAGATATAAAGAATGTTGAC
MBP-SHLD2(1-60)	GGAATTCATGAGTGGAGGATCTCAAGTCC	CCGCTCGAGTCATTCAAGATTTTTGTGCTGTTTTTC

Supplementary Table 2 (continued). Primers for constructing plasmids.

cREV1 CTD-His	GGAATTCCCATGGGCTCTCACAAAAAATCTTTCTTCGACAAAAAACG	CCGCTCGAGTGTAACTTTTAATGTGCTTCCAT
REV3(1871-2021)-His	CATGCCATGGGCACCCCTCGAACTGCTAACATTCT	CCGCTCGAGTTTCTTGGAACGTTCGTATTCTTCT
MBP-REV3(1847-2021)	GGAATTCATGTTGACACCAACTCCTGATAGTT	ATAAGAATGCGGCCGCTCATTTCTTGGAACGTTCGTATTCTTCT
MBP-REV3(1847-1906)	GGAATTCATGTTGACACCAACTCCTGATAGTT	ATAAGAATGCGGCCGCTCATTCCTGGTAAATAGTCTCAGACAGGTC
REV3(1847-1906)-His	CATGCCATGGGCATGCTGACCCCGACCCCGGACTCTTCTCCGCGTTCTACCTCTTCTC	CCGCTCGAGTTCCTGGTAGATGGTTTCAGACAGGTCGTGGTCCAGCAGGGT
GFP-REV3^{TR1}	See below (3 steps)	See below (3 steps)
1st: 1847-3130	TCCGGACTCAGATCTCGAGCTATGTTGACACCAACTCCTGATAG	AGATCCGGTGGATCCTTAAAACTGGTCTAATAACTGCCG
2nd: 1-526	TACAAGTCCGGACTCAGATCTCGAGCTATGTTTTCAGTAAGGATAGTGACTGC	TTGGTGTCAACATAGCGAATTCTCCATCTAACTGAGGTATAGAAAGAC
3rd: 1042-1251	AAGGATGACGACGATAAGCCAAAGAAAAGTCACAGAAGAAAGT	GGAGTTGGTGTCAACATAGCGAATTCAGACACATTCTGGTGTTTC
GFP-REV3^{FL}	TACAAGTCCGGACTCAGATCTCGAGCTATGTTTTCAGTAAGGATAGTGACTGC	AGATCCGGTGGATCCTTAAAACTGGTCTAATAACTGCCG
GFP-REV3(1042-1251+1847-2021)	AAGGATGACGACGATAAGCCAAAGAAAAGTCACAGAAGAAAGT	GGAGGGAGAGGGGCGTTATTTCTTGGAACGTTCGTATTCTT
GFP-REV3(1847-2021)	GGAATTCTATGTTGACACCAACTCCTGATAG	CGGGATCCTCATTTCTTGGAACGTTCGTATTCTT

Supplementary Table 3. Primers for mutagenesis.

Vector	Forward primer (5'-3')	Backward primer (5'-3')
REV7^{E35A}	CTCTACGTGCGCGCGGTCTACCCCGTG	CACGGGGTAGACCGCGCGCACGTAGAG
REV7^{K44A}	CCGTGGGCATCTTCCAGGCACGCAAGAAGTACAACG	CGTTGTACTTCTTGCGTGCCTGGAAGATGCCCACGG
REV7^{Y63A}	CCCGGAGCTGAATCAGGCTATCCAGGACACGCTG	CAGCGTGTCCTGGATAGCCTGATTCAGCTCCGGG
REV7^{R124A}	GGAGCAGCTGCTCGCGGCCTTCATCCTG	CAGGATGAAGGCCGCGAGCAGCTGCTCC
REV7^{K129A}	CGGGCCTTCATCCTGGCGATCAGCGTGTGCGAT	ATCGCACACGCTGATCGCCAGGATGAAGGCCCG
REV7^{W171A}	CATCAAGGATTTCCCCGCGATCCTGGCGGATGAG	CTCATCCGCCAGGATCGCGGGGAAATCCTTGATG
REV7^{R185A}	GTCCACATGCATGACCCCGCGCTGATACCACTAAAAAC	GTTTTTAGTGGTATCAGCGCGGGGTCATGCATGTGGAC
REV7^{K190A}	CCCCCGGCTGATACCACTAGCAACCATGACGTCGG	CCGACGTCATGGTTGCTAGTGGTATCAGCCGGGGG
SHLD3(1-82) ^{F38A}	CGTCCGCTGTCTCGTGCCATCCCGTGGTTCCC	GGGAACCACGGGATGGCACGAGACAGCGGACG
SHLD3(1-82) ^{5A}	CCGACCCGTCCGCTGTCTCGTGCCATCGCGGCGGCCGCGTACGACGGTTCTAAAC	GTTTAGAACCGTCGTACGCGGCCGCCGCGATGGCACGAGACAGCGGACGGGTCGG
REV7-SHLD3(38-82)	GTATAAGAAGGAGATATACATATGTTCATCCCGTGGTTCCCG	CGGGAACCACGGGATGAACATATGTATATCTCCTTCTTATAC
REV7-SHLD3(45-82)	AAGAAGGAGATATACATATGGACGGTTCTAAACTGCCGC	GCGGCAGTTTAGAACCGTCCATATGTATATCTCCTTCTT
