# Supplementary material for: Molecular basis for assembly of the shieldin complex and its implications for NHEJ
Source: Nat Commun. 2020 Apr 24;11:1972. doi: 10.1038/s41467-020-15879-5 (PMC7181697; doi:10.1038/s41467-020-15879-5)
Supplement: Supplementary file 3 — Reporting Summary [file 41467_2020_15879_MOESM3_ESM.pdf]

nature research

Corresponding author(s): Ling Liang and Yuxin Yin

Last updated by author(s): Mar 5, 2020

Reporting Summary

Nature Research wishes to improve the reproducibility of the work that we publish. This form provides structure for consistency and transparency in reporting. For further information on Nature Research policies, see Authors & Referees and the Editorial Policy Checklist.

Statistics

For all statistical analyses, confirm that the following items are present in the figure legend, table legend, main text, or Methods section.

n/a	Confirmed	
☐	☒	The exact sample size (*n*) for each experimental group/condition, given as a discrete number and unit of measurement
☐	☒	A statement on whether measurements were taken from distinct samples or whether the same sample was measured repeatedly
☐	☒	The statistical test(s) used AND whether they are one- or two-sided<br>*Only common tests should be described solely by name; describe more complex techniques in the Methods section.*
☒	☐	A description of all covariates tested
☒	☐	A description of any assumptions or corrections, such as tests of normality and adjustment for multiple comparisons
☐	☒	A full description of the statistical parameters including central tendency (e.g. means) or other basic estimates (e.g. regression coefficient) AND variation (e.g. standard deviation) or associated estimates of uncertainty (e.g. confidence intervals)
☒	☐	For null hypothesis testing, the test statistic (e.g. *F*, *t*, *r*) with confidence intervals, effect sizes, degrees of freedom and *P* value noted<br>*Give P values as exact values whenever suitable.*
☒	☐	For Bayesian analysis, information on the choice of priors and Markov chain Monte Carlo settings
☒	☐	For hierarchical and complex designs, identification of the appropriate level for tests and full reporting of outcomes
☒	☐	Estimates of effect sizes (e.g. Cohen's *d*, Pearson's *r*), indicating how they were calculated

Our web collection on statistics for biologists contains articles on many of the points above.

Software and code

Policy information about availability of computer code

Data collection	X-ray diffraction data were collected at beamline BL18U1 at Shanghai Synchrotron Radiation Facility (SSRF). The diffraction data were processed using HKL2000 and CCP4 (7.0.039).
Data analysis	X-ray data analysis was done using Phaser, Phenix (1.11.1) and COOT (0.8.8). Multi-angle light scattering data analysis was done using the ASTRA 6 software. Isothermal titration calorimetry data analysis was done using the MicroCal software (Origin 7). Additional software including Graphpad Prism 5 (Version 5.01), PISA server v1.52, swiss PDB viewer 4.1, gromacs 2019.1 and imagej 1.51s were used.

For manuscripts utilizing custom algorithms or software that are central to the research but not yet described in published literature, software must be made available to editors/reviewers. We strongly encourage code deposition in a community repository (e.g. GitHub). See the Nature Research guidelines for submitting code & software for further information.

Data

Policy information about availability of data

All manuscripts must include a data availability statement. This statement should provide the following information, where applicable:

- Accession codes, unique identifiers, or web links for publicly available datasets
- A list of figures that have associated raw data
- A description of any restrictions on data availability

Coordinates and structure factors for the crystal structure of human SHLD3-C-REV7-O-REV7-SHLD2 complex have been deposited into the Protein Data Bank with the accession code 6KTO. Structural details about REV7-REV3-REV1 (PDB ID: 3VU7) and C-Mad2-O-Mad2 (PDB ID: 2V64) are accessible in the Protein Data Bank (PDB). A source data file containing raw data for most figures has been provided. All other data that support the study are available from the corresponding authors upon reasonable request.

Field-specific reporting

Please select the one below that is the best fit for your research. If you are not sure, read the appropriate sections before making your selection.

☒ Life sciences ☐ Behavioural & social sciences ☐ Ecological, evolutionary & environmental sciences

For a reference copy of the document with all sections, see nature.com/documents/nr-reporting-summary-flat.pdf

Life sciences study design

All studies must disclose on these points even when the disclosure is negative.

Sample size	Sample size was chosen based on the standard practices in the field. No statistical methods were used to predetermine the sample size.
Data exclusions	No data were excluded.
Replication	All biochemical and cellular assays are repeated at least two times with similar results, unless otherwise stated. The number of replicate for each experiment is shown in the figure legends.
Randomization	Randomization is not applicable for the biochemical and structural work presented. Random fields of vision were analyzed when performing laser micro-irradiation and imaging of live cells.
Blinding	Blinding is not relevant for the structural work presented, and is not necessary as samples were not allocated to groups.

Reporting for specific materials, systems and methods

We require information from authors about some types of materials, experimental systems and methods used in many studies. Here, indicate whether each material, system or method listed is relevant to your study. If you are not sure if a list item applies to your research, read the appropriate section before selecting a response.

Materials & experimental systems

n/a	Involved in the study	
☐	☒	Antibodies
☐	☒	Eukaryotic cell lines
☒	☐	Palaeontology
☒	☐	Animals and other organisms
☒	☐	Human research participants
☒	☐	Clinical data

Methods

n/a	Involved in the study	
☒	☐	ChIP-seq
☒	☐	Flow cytometry
☒	☐	MRI-based neuroimaging

Antibodies

Antibodies used	The following antibodies were used for immunoblotting: anti-Flag (Sigma-Aldrich, F3165), anti-HA (Sigma-Aldrich,H3663) and anti-GFP (Ray Antibody, RM1008).
Validation	Monoclonal anti-Flag (Sigma-Aldrich, F3165) produced in mouse has been validated in immunoblotting, immunoprecipitation, immunocytochemistry, immunofluorescence, ELISA, chromatin immunoprecipitation, electron microscopy, flow cytometry and supershift assays on the manufacturer's website (www.sigmaaldrich.com). Monoclonal Anti-HA antibody (Sigma-Aldrich,H3663) produced in mouse has been validated in immunoblotting, immunocytochemistry and immunoprecipitationon on the manufacturer's website (www.sigmaaldrich.com). Monoclonal anti-GFP (Ray Antibody, RM1008) produced in mouse has been validated in immunoblotting and immunoprecipitationon by Guoliang Li et al., Autophagy, 2018 (PMID: 29969932).

Eukaryotic cell lines

Policy information about cell lines

Cell line source(s)	HEK293FT cells were obtained from Thermo Fisher Scientific. HEK293T and HeLa cells were obtained from the American Type Culture Collection (ATCC).
Authentication	HeLa cell line has been validated to be HeLa origin by STR profiling. HEK293T and HEK293FT cell line was not authenticated.
Mycoplasma contamination	All of the cell lines are negative for mycoplasma. We routinely check the cell lines for mycoplasma contamination by Hoechst staining.

Commonly misidentified lines (See ICLAC register)	Hela, HEK293T and HEK293FT cells are not commonly misidentified lines.
